# The significance of biopsy scar excision at the time of skin- or nipple-sparing mastectomy with immediate breast reconstruction

**DOI:** 10.1093/jjco/hyab065

**Published:** 2021-05-04

**Authors:** Yuri Ozaki, Akiyo Yoshimura, Masataka Sawaki, Masaya Hattori, Haruru Kotani, Yayoi Adachi, Ayumi Kataoka, Kayoko Sugino, Nanae Horisawa, Yuka Endo, Kazuki Nozawa, Shoko Sakamoto, Daiki Takatsuka, Seiko Okumura, Yoko Maruyama, Hiroji Iwata

**Affiliations:** Department of Breast Oncology, Aichi Cancer Center, Nagoya, Japan; Department of Breast Oncology, Aichi Cancer Center, Nagoya, Japan; Department of Breast Oncology, Aichi Cancer Center, Nagoya, Japan; Department of Breast Oncology, Aichi Cancer Center, Nagoya, Japan; Department of Breast Oncology, Aichi Cancer Center, Nagoya, Japan; Department of Breast Oncology, Aichi Cancer Center, Nagoya, Japan; Department of Breast Oncology, Aichi Cancer Center, Nagoya, Japan; Department of Breast Oncology, Aichi Cancer Center, Nagoya, Japan; Department of Breast Oncology, Aichi Cancer Center, Nagoya, Japan; Department of Breast Oncology, Aichi Cancer Center, Nagoya, Japan; Department of Breast Oncology, Aichi Cancer Center, Nagoya, Japan; Department of Breast Oncology, Aichi Cancer Center, Nagoya, Japan; Department of Breast Oncology, Aichi Cancer Center, Nagoya, Japan; Department of Plastic Surgery, Aichi Cancer Center, Nagoya, Japan; Department of Plastic Surgery, Aichi Cancer Center, Nagoya, Japan; Department of Breast Oncology, Aichi Cancer Center, Nagoya, Japan

**Keywords:** breast cancer, biopsy scar, neoplastic seeding, breast reconstruction

## Abstract

**Background:**

Neoplastic seeding (NS) can occur after tissue biopsy, which is a clinical issue especially in mastectomy with immediate reconstruction. This is because postoperative radiation is not usually given and local recurrence of preserved skin flap may increase. The purpose of this study is to investigate the importance of preoperative evaluation of NS and the validity of biopsy scar excision.

**Patients and methods:**

We retrospectively analysed 174 cases of mastectomy with immediate breast reconstruction. The primary endpoint is the frequency of clinical and pathological NS and the secondary endpoint is the problem of excision of needle biopsy site.

**Results:**

Three cases (1.7%) had preoperative clinical findings of NS. Pathological examination revealed NS in all three cases. Biopsy scars could be excised in 115 cases among 171 cases without clinical NS. Pathological NS was found in 1 of 66 (1.5%) cases of which pathological examination was performed. Biopsy scars could not be excised in the remaining 56 cases: the biopsy scar could not be identified in 41 cases, and there was concern about a decrease in flap blood flow after excision in 15 cases. In 12 of these 15 cases, the scars were close to the skin incision; excision of these scars might have triggered skin necrosis between the incision and the biopsy scar excision site. No postoperative complications were observed.

**Conclusions:**

It is important to preoperatively evaluate clinical NS, and biopsy scars should be excised in clinical NS cases. Even in cases without clinical NS, biopsy scar excision should be considered. It is also important to perform a biopsy in consideration of the incision design for reconstructive surgery.

## Teaser

It is important to evaluate clinical neoplastic seeding (NS) due to biopsy. Biopsy scars should be excised in clinical NS cases. Excision should be considered even without clinical NS.

## Introduction

Pathological diagnosis by percutaneous image-guided biopsy is essential for breast cancer treatment. Although fine needle aspiration cytology has been used extensively, core needle biopsy (CNB) and vacuum-assisted biopsy (VAB) are recommended for more accurate diagnosis. Furthermore, they facilitate biomarker evaluation, which is critical for determining the choice of primary systemic therapy (1,2).

However, it is a clinical issue of CNB and VAB that neoplastic seeding (NS) can occur along the needle track. The frequency of NS varies widely (0.2–69%) between previous studies (3–10). One of the explanations for this considerable variation is that these studies used different methods to evaluate NS, including core wash cytology (8,10) and evaluation of NS only in cases where it was suspected as following image diagnosis (4). It has been also shown that the frequency of confirmed disseminated cancer cells decreases with the passage of time until surgery (3). High histological grade (4), triple negative (4), use of CNB rather than VAB (3,10), multiple puncture (3,4,7,8) and ductal carcinoma rather than lobular carcinoma (3,8,10) have been reported as risk factors for NS.

Local recurrences to a remaining biopsy scar have been reported in cases after mastectomy without breast reconstruction, where the largest amount of skin and subcutaneous tissue is removed among breast cancer surgery (11,12). In the case of breast-conserving surgery, the risk of local recurrence due to the remaining biopsy scar and part of the needle tract could be higher, but postoperative whole-breast irradiation is thought to control local recurrence (9,13–15). In the case of mastectomy with immediate reconstruction, postoperative radiation is not usually performed despite of preserving the skin. Thus, there is concern of a higher risk of local recurrence to the overlying skin flap due to NS. Although some studies have reported cases of local recurrence in biopsy scar after skin-sparing mastectomy (16–18), it remains unclear whether the biopsy scar should be excised. In this study, we focus on the importance of preoperative evaluation of NS and the validity of biopsy scar resection at the time of skin- or nipple-sparing mastectomy with immediate breast reconstruction.

## Patients and methods

### Patients

This study includes 174 breast cancer patients who were diagnosed using CNB or VAB and who underwent skin- or nipple-sparing mastectomy with immediate breast reconstruction at our hospital between August 2018 and April 2020.

### Biopsy procedures

Forty-eight cases biopsied at our hospital were all diagnosed with VAB using 11G Mammotome® EX (Devicor Medical Products, Inc., USA), which allows multiple samples with a single puncture. In the remaining 128 cases biopsied at other hospitals, the physician chose CNB or VAB for diagnosis.

### Preoperative clinical findings

Preoperative examinations including physical examination, mammography and ultrasonography were performed in all cases, and MRI and CT examinations were performed on a case-by-case basis. All patients underwent physical examination and ultrasonography for final confirmation after being hospitalized for surgery. The biopsy scar was examined for redness and induration by physical examination. Masses along the biopsy tract were identified using ultrasonography. These findings were defined as clinical NS of this study and the presence or absence of these findings for all reconstructive surgery were recorded.

### Surgical procedures and pathology

**Figure 1.**
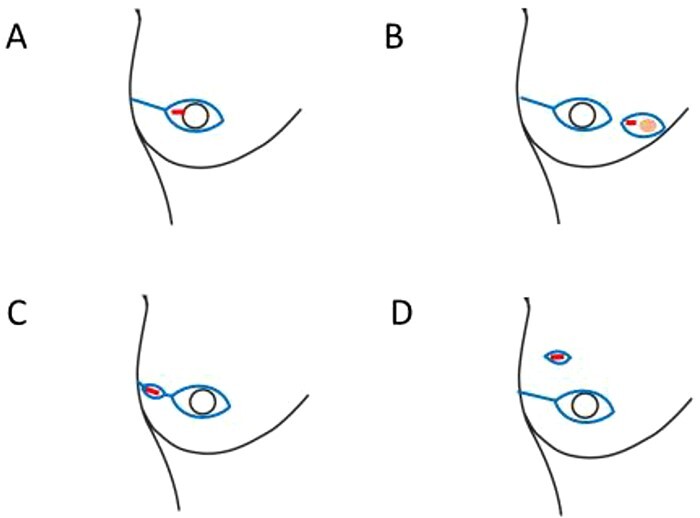
Biopsy scar excision techniques depending on the location of the biopsy scar. A. combined excision with the nipple areola. B. combined excision with the skin above the tumour. C. excision on the line of the oblique incision. D. excision of only the biopsy scar by additional skin incision.

We usually performed skin- or nipple-sparing mastectomy by oblique incision with or without nipple areola resection in principle. Depending on the location of the biopsy scar, it was excised using one of the following four techniques: (A) combined excision with the nipple areola, (B) combined excision with the skin above the tumour, (C) excision on the line of the oblique incision and (D) excision only the biopsy scar by making an additional skin incision (Fig. 1). We intraoperatively evaluated blood flow within the overlying skin flap using indocyanine green dye (ICG) fluorescence imaging in all cases of breast reconstruction. Technique D was performed only when the blood flow was sufficient and it was determined that additional excision did not adversely affect flap blood flow. These decisions were made jointly by breast surgeons and plastic surgeons. All excised biopsy scars were submitted for pathological examination.

### Adverse events and safety

Postoperative complications due to the biopsy scar excision, including skin ulceration and skin infection, were evaluated using Common Terminology Criteria for Adverse Events (CTCAE) Version5.0.

### Assessments

The primary endpoint of this study is the frequency of clinical and pathological NS. Clinical NS was defined as redness and induration at the biopsy scar by physical examination and/or masses along the biopsy tract identified by any image diagnosis. Pathological NS was defined as the presence of cancer microscopically in the skin and subcutaneous tissue of the biopsy scar. The secondary endpoint is the problem of excision of needle biopsy site at the time of immediate reconstruction, including the reason for not excising the biopsy scar and postoperative complications.

### Ethics approval and consent to participate

All procedures performed involving human participants were in accordance with the ethical standards of the institutional review board of Aichi Cancer Center. Informed consent was obtained from the patient for this publication.

## Results

**Figure 2.**
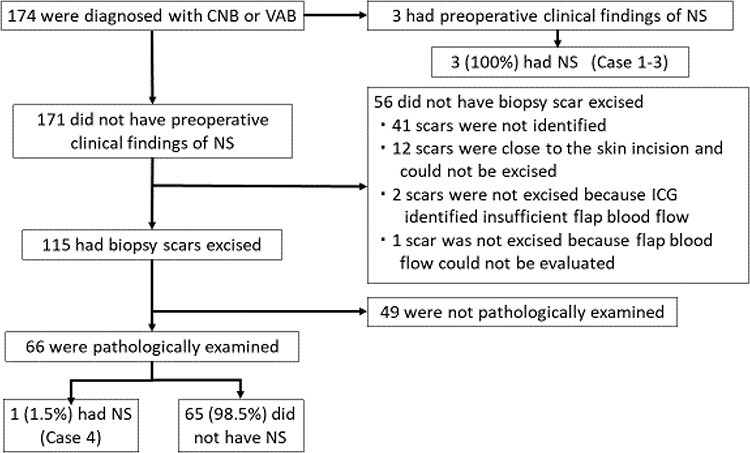
Study profile. CNB: core needle biopsy. VAB: vacuum-assisted biopsy. NS: neoplastic seeding. ICG: indocyanine green angiography imaging.

**Figure 3.**
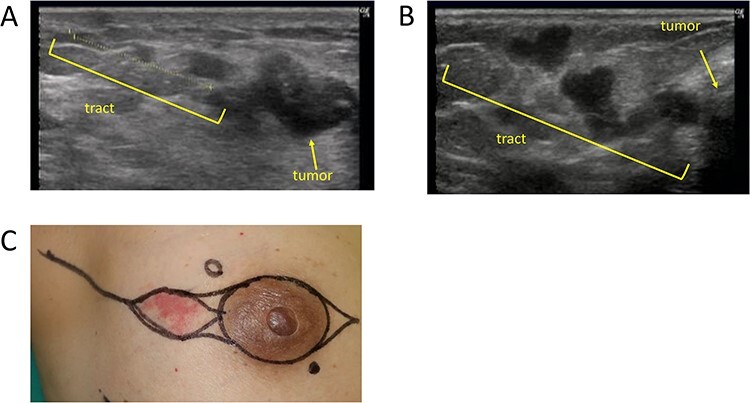
Clinical findings of Cases 1–3. A and B show multiple masses along the needle tract in cases 1 and 2. C shows a red flare on the biopsy scar in case 3.

**Figure 4.**
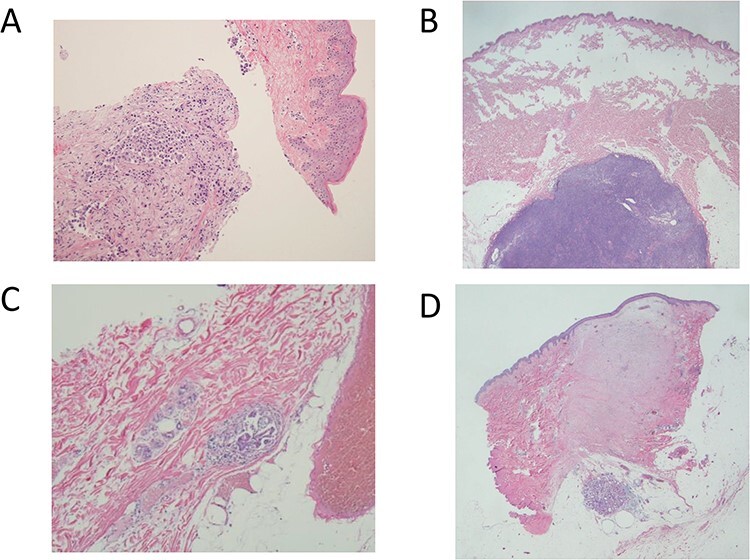
Pathological findings of case 1–4. A, B and D show invasive cancer just below the biopsy scar in cases 1, 2 and 4, respectively. C shows microinfiltration lesions in the dermis of the biopsy scar in case 3.

The mean age was 49 years old (range 30–77 years old). Surgical procedures are shown in Table 1. A clinical diagnosis of NS was made in three cases (1.7%) (Fig. 2). Cases 1 and 2 had multiple masses along the needle tract (Fig. 3a and b). In case 1, the biopsy was performed by the previous physician and the patient was referred to our hospital for surgery. Preoperative reassessment at our hospital revealed tumour formation along the tract. In case 2, tumour progression was observed during preoperative chemotherapy; concomitantly, tumour formation along the tract was also observed. Case 3 had red flare on the biopsy scar (Fig. 3c). In this case, the flare appeared around the biopsy scar after administration of the last round of preoperative chemotherapy. The scars in cases 1, 2 and 3 were excised by additional incision (excision technique D), on the line of the oblique incision (excision technique C) or in combination with the nipple areola (excision technique A), respectively. Pathological analysis revealed NS in all three cases (Fig. 4a-c). In the two cases in which multiple masses along the tract were found using ultrasonography (cases 1 and 2), pathological NS was observed subcutaneously just below the biopsy scar and along the tract as shown in the image. In the case with red flare on the biopsy scar (case 3), microinfiltration lesions were found in the dermis of the scar. All cases were histological grade 3 (Table 2). All presented with lymphvascular invasion, which was severe in two cases. Cases 1 and 2 were invasive ductal carcinoma, and case 3 was invasive micropapillary carcinoma. Case 2 had no pathological therapeutic effect on neoadjuvant chemotherapy. On the other hand, there was no obvious tendency in the biopsy procedure and the days from biopsy to surgery (Table 2).

**Table 1:** Surgical procedures (*n* = 174)

Breast surgery	
Skin-sparing mastectomy	150
Nipple-sparing mastectomy	24
Axilla surgery	
Sentinel lymph node biopsy	149
Axillary dissection	25
Breast reconstruction surgery	
Immediate one-stage IMP	78
Immediate expander insertion	11
Immediate one-stage LD	50
Immediate one-stage TRAM	35

IMP, implant breast reconstruction; LD, latissimus dorsi flap reconstruction; TRAM, transverse rectus abdominis musculocutaneous flap.

**Table 2:** Patient characteristics

	Cases with clinical and pathological NS (Cases 1–3)	Case without clinical NS but with pathological NS (Case 4)	Cases without clinical and pathological NS (*n* = 65)
Biopsy procedure			
US-guided CNB	2	0	27
—median number of samples (range)	3.5 (2–5) samples		4 (1–9)^b^ samples
US-guided VAB	1	1	31
Stereotactic VAB	0	0	7
Neoadjuvant chemotherapy			
Yes	2	0	8
—median days from biopsy to surgery (range)	211 (104–214) days		212 (147–292)^c^ days
No	1	1	57
—median days from biopsy to surgery (range)	93 days	71 days	98 (47–162)^d^ days
Histology			
DCIS	0	0	10
IDC	2	1	47
ILC	0	0	6
Invasive micropapillary carcinoma	1	0	0
Others	0	0	2
Histological grade			
1	0	1	21
2	0	0	24
3	3	0	10
Lymph invasion			
Yes	3	1	34
No	0	0	31
Vascular invasion			
Yes	3	1	8
No	0	0	57
Subtype^a^			
Luminal A	0	1	25
Luminal B	1	0	13
Luminal-HER2	1	0	7
Pure HER2	1	0	6
Triple negative	0	0	4

US, ultrasonography; CNB, core needle biopsy; VAB, vacuum-assisted biopsy; NS, neoplastic seeding; DCIS, ductal carcinoma in situ; IDC, invasive ductal carcinoma; ILC, invasive lobular carcinoma.

^a^Invasive carcinoma. Luminal A: estrogen recepter (ER)-positive (Allred score ≥ 3), human epidermal growth factor type 2 (HER2)-negative, Allred of ER + progesterone receptor (PR) ≥13, histological grade 1 or 2, and Ki67 ≤ 20%. Luminal B: ER-positive and HER2-negative, excepting luminal A. Luminal- HER2: ER-positive and HER2-positive. Pure HER2: ER-negative and HER2-positive. Triple negative: ER-negative and HER2-negative.

^b^Four unknown cases.

^c^One unknown case.

^d^Three unknown cases.

Of the 171 cases without clinical NS, biopsy scars were excised in 115 cases (Fig. 2). Sixty could be excised with an incision scheduled for breast cancer surgery (excision techniques A–C). The remaining 55 were excised by making an additional incision after confirming sufficient flap blood flow by ICG fluorescence imaging (excision technique D). Pathological NS was found in 1 of 66 cases (1.5%) in which pathological examination was performed (Fig. 2). This patient (case 4) had invasive cancer in the subcutaneous tissue just below the biopsy scar. (Fig. 4d). The case was histological grade 1 and luminal A. Lymphvascular invasion was observed, but no other obvious risk factors could be identified (Table 2).

We could not excise the biopsy scar in 56 cases (Fig. 2). Biopsy scars could not be identified at the time of surgery in 41 cases. We could not excise biopsy scars in 12 cases (6.9%) despite sufficient overlying flap blood flow, because the scars were close to the skin incision that was made during breast cancer surgery. Excision of such scars could trigger necrosis in the skin between the skin incision and the biopsy scar excision site. Two cases had insufficient flap blood flow according to ICG fluorescence imaging, making it impossible to create an additional incision for biopsy scar excision. We could not excise one biopsy scar by making an additional skin incision, because flap blood flow could not be evaluated by ICG fluorescence imaging due to suspected allergy to ICG.

No postoperative complications due to biopsy scar excision (including skin ulceration or skin infection) were observed. No local recurrence has occurred at the residual biopsy scars or at the biopsy scar excision site over a median observation period of 16.9 months (2.6–27.2 months).

## Discussion

It is a clinical issue that NS can occur after CNB or VAB, although these percutaneous image-guided biopsies play an essential role in breast cancer treatment for accurate diagnosis and biomarker evaluation (1,2). To our knowledge, no studies have focused on the incidence of NS in patients who have undergone breast reconstruction. Our study thus provides valuable information regarding biopsy scar excision in clinical practice.

In this study, we found that the frequency of clinical NS detected after preoperative physical examination and ultrasonography was 1.7%. A similar retrospective study reported that 8 of 4010 cases were suspected as clinical NS-positive (4). Seven of the eight cases were found preoperatively by diagnostic imaging, and the remaining one was found as postoperative palpable mass, meaning that preoperatively suspected NS accounted for 0.17% of all cases (7/4010). To find out why the frequency of our clinical NS was higher, differences in background such as the definition of clinical NS and days from the biopsy. In our study, cases with redness, induration and masses along the tract were defined as clinical NS and were recorded in all reconstructive surgery. In the previous reports, the electric database was searched for the following descriptors: *tumor progression, disease progression, recurrence, bracketing and seeding*. Although it is difficult to make simple numerical comparisons because the definitions are different, it is possible that our study more accurately detected the frequency of NS-specific clinical findings. On the other hand, the days from the biopsy to NS diagnosis in our cases were 93, 194 and 214 days, and the mean days of the previous study were 60.8 days. Daiz et al. showed that the rate of confirmed disseminated cancer cells decreased with the passage of time until surgery and therefore hypothesized that malignant cells present in the tract immediately after the biopsy cannot survive for long periods (3). From this theory, the number of days from biopsy to surgery has little relevance to the frequency of NS of our study. The difference in frequency may also be related to the small number of cases in our study or to the fact that our reassessment was carried out just before surgery.

The pathological characteristics of our clinical NS cases were high tumour malignancy such as histological grade 3, lymphvascular invasion or chemotherapy refractory. On the other hand, we were unable to find a clear association between the biopsy procedure and NS. Clinical NS that is large enough to be obvious on physical examinations and images may be associated with the tumour malignancy itself rather than with the biopsy procedure. NS was pathologically observed in all of three cases with clinical NS, highlighting the importance of preoperative evaluation and the necessity to excise biopsy scar in all cases with clinical findings.

As far as we are aware, there are three reports that looked for pathological NS in surgical specimens from patients with no actual clinical signs of NS (3,7,9). Hoorntje et al. performed biopsy and surgery on the same day and reported that NS along the needle tract was seen in 11/22 cases (50%) (9). The reason for this high frequency is that the entire tract was evaluated in addition to the biopsy scar, and the biopsy and surgery were on the same day. Stolier et al. examined only biopsy scars and observed pathological NS in 2/89 (2.2%) with an average biopsy to surgery days of 10.5 (7). Our finding is consistent with this previous study, as pathological NS was observed at a frequency of 1.5%, considering our long period from biopsy to surgery. In our case, we identified lymphvascular invasion as a potential risk factor but did not find any of the other previously reported risk factors such as specific pathological characteristics and biopsy procedure. In other words, it may be difficult to predict a case with pathological NS. On the other hand, we did not observe any complications due to biopsy scar excision. For these reasons, biopsy scar excision may be considered even in cases where there are no signs of clinical NS.

In skin- or nipple-sparing mastectomy, the entire glands are removed and most of the skin is conserved to create a pocket that is filled with a breast implant or the patient’s own tissue. Necrosis of the overlying skin flap is a major surgical complication. It may cause infection and subsequent removal of the implant or cause scar contracture, which leads to a poor cosmetic outcome. Adding an additional incision to the skin flap for biopsy scar excision is expected to increase the risk of necrosis, which is not a problem in cases where mastectomy is performed without reconstruction or breast-conserving surgery. ICG fluorescence imaging can be useful in predicting skin flap necrosis (19). When the blood flow of the skin flap is determined to be insufficient, a biopsy scar cannot be excised by making an additional incision. Even when the blood flow is sufficient, a biopsy scar that is close to the site of skin incision for breast cancer surgery cannot be excised, because additional incision would impair blood flow between the incisions. For this reason, we were unable to excise biopsy scars in 12 cases (6.9%). In these cases, it is possible that the biopsy scars could have been safely excised if the biopsies were performed considering the skin incision design for reconstructive surgery.

This study has a few limitations. First, we studied only a limited number of cases. In particular, 49 of the 115 (42.6%) biopsy scars without clinical NS were not pathologically examined for NS. In these cases, it was difficult to evaluate the pathology of biopsy scars excised on the line of the oblique incision, or those with involvement of the nipple areola or the skin above the tumour. Second, not all seeded cells in residual biopsy scars translate into local recurrence. Moreover, even if local recurrence occurs at the biopsy scar, it may be possible to detect the recurrence by physical examination and promptly perform a resection. Therefore, it is unclear whether biopsy scar excision contributes to the improved survival rate in cases without clinical NS. However, we suggest that biopsy scar excision should always be considered, as it is a relatively low-risk procedure.

In summary, it is important to evaluate clinical NS preoperatively by physical examination and ultrasonography. Although biopsy scars should be excised in clinical NS cases where possible, we also recommend that excision should be considered even in cases without clinical NS. It is also important that skin incision design be carefully planned prior to performing a biopsy, as this will permit the future excision of biopsy scars if required.

## Conflict of Interest

Nothing to declare.
